# Fungal Mycobiome of Mature Strawberry Fruits (*Fragaria x ananassa* Variety ‘Monterey’) Suggests a Potential Market Site Contamination with Harmful Yeasts

**DOI:** 10.3390/foods13081175

**Published:** 2024-04-12

**Authors:** Gabriela N. Tenea, Pamela Reyes, Diana Molina

**Affiliations:** Biofood and Nutraceutics Research and Development Group, Faculty of Engineering in Agricultural and Environmental Sciences, Universidad Técnica del Norte, 100150 Ibarra, Ecuador

**Keywords:** strawberries, ITS2 metabarcoding, *Kurtzmaniella* spp., *Candida parapsilosis*, *Cryptococcus* spp.

## Abstract

An amplicon metagenomic approach based on the ITS2 region of fungal rDNA was used to investigate the diversity of fungi associated with mature strawberries collected from a volcanic orchard and open-air market stands. Based on the Kruskal–Wallis test, no statistically significant differences were observed in both non-phylogenetic and phylogenetic alpha diversity indices. According to beta diversity analyses, significant differences in fungal communities were found between groups (orchard vs. market). Taxonomic assignment of amplicon sequence variables (ASVs) revealed 7 phyla and 31 classes. The prevalent fungal phyla were *Basidiomycota* (29.59–84.58%), *Ascomycota* (15.33–70.40%), and Fungi-phy-Insertae-sedis (0.45–2.89%). The most predominant classes among the groups were *Saccharomycetes* in the market group, and *Microbotryomycetes* and *Tremellomycetes* in the orchard group. Based on the analysis of microbiome composition (ANCOM), we found that the most differentially fungal genera were *Hanseniaspora*, *Kurtzmaniella*, and *Phyllozyma.* Endophytic yeasts *Curvibasidium cygneicollum* were prevalent in both groups, while *Candida railenensis* was detected in fruits originating only from the market. In addition, *Rhodotorula graminis* (relative abundance varying from 1.7% to 21.18%) and *Papiliotrema flavescens* (relative abundance varying from 1.58% to 16.55%) were detected in all samples regardless of origin, while *Debaryomyces prosopidis* was detected in samples from the market only, their relative abundance varying with the sample (from 0.80% to 19.23%). Their role in fruit quality and safety has not been yet documented. Moreover, several clinically related yeasts, such as *Meyerozyma guilliermondii* and *Candida parapsilosis*, were detected in samples only from the market. Understanding the variety and makeup of the mycobiome in ripe fruits during the transition from the orchard to the market is crucial for fruit safety after harvest.

## 1. Introduction

The berries most consumed, strawberries (*Fragaria x ananassa* Duch.), are non-climatic and highly perishable fruits, vulnerable to phytopathogen infection and extremely sensitive to postharvest storage [1]. Strawberries have a very short postharvest self-life (about seven days at 4 °C and three days at room temperature) [2]. The loss of weight and firmness is the main problem, in addition to the change in its organoleptic characteristics [2]. Healthy strawberries have a diverse microbiota that includes molds potential plant pathogens, and human pathogens [3]. The airborne fungus causes the deterioration of fruits and loses marketability [4]. To reduce fungus infections, fungicides are applied; however, their overuse and improper application have several drawbacks, such as risks to human health, adverse effects on nontarget organisms, the environment, and the emergence of resistant genotypes [5]. Due to their widespread presence in the environment, fungi are crucial parts of both agricultural and natural ecosystems, where they are essential for the turnover of nutrients [6]. Fruit-associated fungi can influence the quality of products derived from horticultural systems and lead to either beneficial or spoiling qualities [7]. 

The microorganisms in fresh fruits have been studied mainly to detect pathogenic bacteria and their possible link to disease outbreaks in humans [8,9]. In addition, fruit damage is likely to occur during handling, uncontrollable harvesting techniques, storage, and transportation conditions, especially when there are large distances between production and market areas [10]. According to early reports, 65% of strawberry loss is related to the transportation from the field to the market [2]. The fungal mycobiome linked to various strawberry organs (leaves, flowers, immature, and mature fruits), which were grown on a farm with regular application of chemical pesticides, was previously disclosed [11]. According to this study, the two most prevalent genera were *Botrytis* and *Cladosporium*, accounting for 70–99% of the relative abundance of all sequences. Agriculture practices and continental location may also influence the global plant microbiome [12]. However, the microbiome associated with strawberries is variety-specific, influenced by management, and related to plant-soil feedback in the phyllosphere and rhizosphere [13]. Moreover, the microbiome of fruits influences the shelf-life [14]. According to early research, fungi belonging to the genera *Botrytis*, *Penicillium*, *Phytophthora*, *Verticillium*, *Alternaria*, *Cladosporium*, *Aureobasidium*, *Cryptococcus*, and *Rhizopus* are the most prevalent pathogens that affect strawberries [15]. *Botrytis cinerea* (gray mold) is considered one of the primary causes of post-harvest losses.

The strawberry growing farms in Northern Ecuador are located at an elevation of 2300 to 2500 m, with primarily volcanic soil. Daytime temperatures range from 18 to 25 degrees Celsius, while nighttime lows are between 8 and 13 degrees Celsius with a relative humidity of 60%. Additionally, farmers are not trained in the use of agrochemicals; this contributes to the empiricism of the region in managing strawberry production [16]. Recently, using 16S DNA metabarcoding analysis, we investigated the bacterial diversity in strawberry fruits from a farm producer and fruits purchased form the market and detected several pathogenic bacteria in the fruit exocarp [17]. However, information on the fungal makeup of strawberry fruits originating from orchards and open-air market sites is scarce. To our knowledge, no research has thoroughly compared the fungal and yeast communities linked to *Fragaria x ananassa* variety ‘Monterey’ fruits collected from a volcanic orchard and commercial stands at ready-to-eat maturity stage. We hypnotized that the fruits at the same maturity stage share similar mycobiome community. Therefore, using an ITS2-based metagenomic approach, we examined the general fungal and budding yeast communities in the exocarp of strawberries harvested from a volcanic orchard (ripe phase four) and different open-air market stands. The mycobiome provide a means of improving fruit safety and aids in the development of additional preventive measures against postharvest contamination.

## 2. Materials and Methods

### 2.1. Fruit Sampling and Processing

Strawberry (*Fragaria x ananassa* variety ‘Monterey’) fruits were collected from a local orchard and different commercial stands of Imbabura Province, Northern Ecuador (geographical coordinates 0°14′00″ N 78°16′00″ O). Fruits in the maturity stage (ready to eat, stage four) with no visible damage and uniform size were harvested from the orchard as follows: 15 fruits per row × 6 field rows × 3 repetitions (total of 270 fruits) during February–October 2023. Commercial fruits were purchased from 6 distinct open-air market stands: 15 fruits × 6 stands × 3 repetitions (total 270 fruits). After being transported to the lab, the fruits were placed in Ziplock bags with 150 mL of sterile peptone water (0.1%). The bags were then incubated for 1.30 h at 37 °C, being gently mixed manually every 30 min. Before DNA extraction, cells surrounding the skin of the fruit (exocarp) were collected by centrifugation at 8000× *g* for 5 min, recovered in 1× PBS, and stored at 4 °C. Genomic DNA was isolated using a commercial column-based ZymoBIOMICS DNA miniprep kit (# D4304, Ecogen Barcelona, Spain) [18].

### 2.2. Library Construction, Sequencing, Data Processing, and Analysis

Illumina Novaseq platform (paired-end 150-bp reads, Illumina, San Diego, CA, USA) was used for metagenomic sequencing [18]. The ITS2 region was amplified using universal fungal primers ITS 86F (5′-GTGAATCATCGAATCTTTGAA-3′) and ITS 4 (5′-TCCTCCGCTTATTGATATGC-3′) [19]. All polymerase chain reaction (PCR) reactions involved the KAPA HiFi HotStart ReadyMix (# 2GFHSRMKB, Sigma Aldrich, St. Louis, MI, USA). A washing step with magnetic beads was used to remove free primers and primer dimers from the amplicons. For sequencing, up to 96 libraries were pooled using all Nextera XT indices. The process of preparing the library, purification, and sequencing was carried out as described [18]. To ensure taxonomic classification, a quality and filtering procedure was applied to the FASTq files. The QIIME v.2 pipeline (Quantitative Insights into Microbial Ecology) [20] was used for the analysis of marker gene-based microbiome sequencing data. The ITS2 region was extracted after the sequences were denoised using the denoise wrapper [21]. Denoising and clustering were performed with DADA2 [22,23]. Chimeric sequences were identified and filtered using USEARCH 6.1 [24]. In addition, a taxonomy is assigned to the sequences. Furthermore, the query ASV sequences were compared with a reference database UNITE Fungal ITS Database v7.2 (https://doi.org/10.15156/BIO/587475, accessed on 14 February 2024). Sequences belonging to chloroplasts, mitochondria, and eukaryotes were removed.

### 2.3. Rarefaction Curves

Rarefaction curves were used to assess how species diversity varied according to the number of sequences (reads) examined in a sample [25].

### 2.4. Analysis of Diversity

Alpha and beta diversity metrics were analyzed as previously described [17]. Briefly, for alpha diversity, the metrics listed below were calculated: (a) Faith’s phylogenetic diversity, a qualitative measure of community richness that considers phylogenetic relationships between traits; (b) Evenness/Pielou uniformity, an index that measures diversity in addition to species richness; (c) Shannon’s diversity index, to estimate species diversity within groups [26]; (d) observed characteristics, a qualitative measure of community wealth. Box plot figures were used to display the results. The alpha diversity data were compared using either a one-way analysis of variance in rank or a nonparametric Kruskal–Wallis test to see if the samples came from the same distribution. Both phylogenetic (UniFrac distance) and non-phylogenetic (Bray–Curtis dissimilarity and Jaccard distance) techniques were applied to measure beta diversity [27]. The statistical significance of the observed differences was assessed using weighted and unweighted UniFrac distance matrices and 999 Monte Carlo permutations. The composition of the fungal community was correlated with the sample group using principal coordinates analysis (PCoA) using QIIME v.2 [20]. Scatter diagrams, either two- or three-dimensional, can be used to visualize the summary of beta diversity relationships. For all statistical procedures, a significance level of 0.05 was considered.

### 2.5. Statistical Significance Tests

The analysis of similarities (ANOSIM) was used to test the hypothesis that there is no difference between the two groups of samples. The R test was employed to determine whether there are differences between the groups under the null hypothesis [28]. Additionally, to infer absolute microbial abundances, the taxa with significantly differential abundances were identified using ANCOM analysis [29]. A centered log ratio (CLR) transformation was applied to account for the compositionality of the microbiome data. The output W statistic indicates how many CLR-transformed models exist for a particular taxon in which the taxon is differentially abundant with the variable of interest. As the W value increases, the probability of a taxa being differentially abundant increases. To ascertain the similarities between the groups at the genus level, hierarchical clustering was carried out using the unweighted pair group method (UPGMA with Euclidean distance). In addition, Venn diagrams were drawn to investigate the intersection of the fungal genera and species between groups (orchard vs. market). A bioinformatic platform (https://www.bioinformatics.com.cn/en, accessed on 14 February 2024) was used to conduct these analyses.

## 3. Results and Discussion

### 3.1. Fungal Communities in Ripe Strawberry Fruits: Alpha-Diversity Analysis

**Figure 1:**
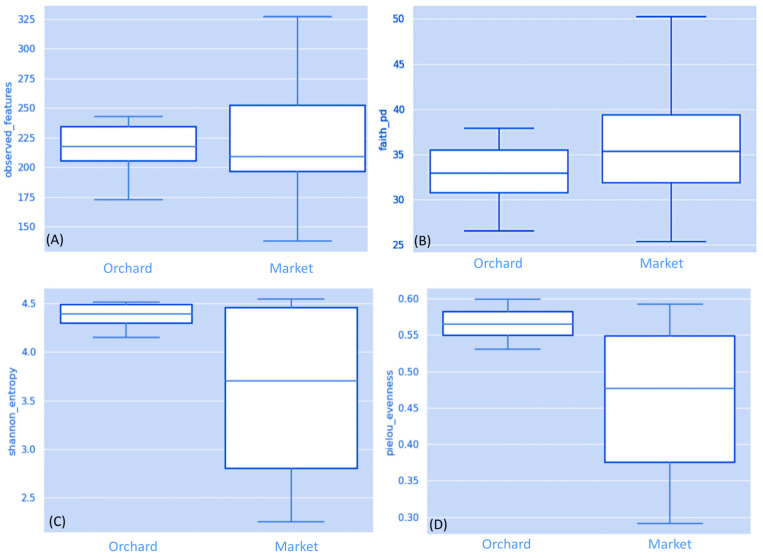
Alpha diversity among groups. Boxplots visualizing results of the nonparametric Kruskal–Wallis to compare the Observed features (**A**); Faith’s PD (**B**); Shannon diversity index (**C**); Pielou’s evenness (**D**). Legend: four: fruits collected from the orchard phase four; market: fruits purchased from the market stands.

Amplicon sequencing was produced from 158,184 to 401,669 high-quality reads per sample (Table S1). Following filtration to eliminate non-target reads (such as mitochondria or chloroplasts), the sequences were attributed to ASVs (Table 1). According to the rarefaction analysis, most of the taxa included in the samples were adequately captured at a sequencing depth of 150,000 (Figure S1). The alpha diversity analysis indicated that strawberry fruits from the market and the orchard share similar fungi diversity (Table 1). Based on the Kruskal–Wallis test, no statistically significant differences were noted in the non-phylogenetic and phylogenetic alpha diversity indices, such as observed ASVs (H = 0.0064; *p* = 0.93), Faith’s PD (H = 0.4102; *p* = 0.52), and Shannon’s index (H = 0.6410; *p* = 0.42) (Figure 1A–D). However, based on Pielou’s evenness index, the samples from the market sites showed a more abundant fungi community than the samples from the orchard (H = 2.5641; *p* = 0.10). In previous research, we showed a significant difference in bacterial alpha diversity in strawberry samples collected from the market at the same ripe stage [17]. Furthermore, according to conventional bacteriological methods conducted in strawberry fruits purchased from the market and orchard at the same ripe stage, a high number of molds and yeasts were detected in the fruit samples from the market [30]. Previously, a metagenomic survey on fungal diversity conducted on different strawberry organs showed high fungal diversity in plant organs (leaves and flowers) rather than immature and mature fruits [11]. Furthermore, the fungal diversity in ripe and stored strawberry fruits was higher than in diseased fruits [31]. Based on 26S ribosomal DNA analysis in ripe greenhouse strawberries, it has been shown that microorganism concentration increased with mechanical damage to the fruit, and its firmness is negatively correlated with the increase of the microorganism in the skin of the fruit [2].

### 3.2. Differential Fungal Community of Ripe Strawberry Fruits: Beta-Diversity Analysis

**Figure 2:**
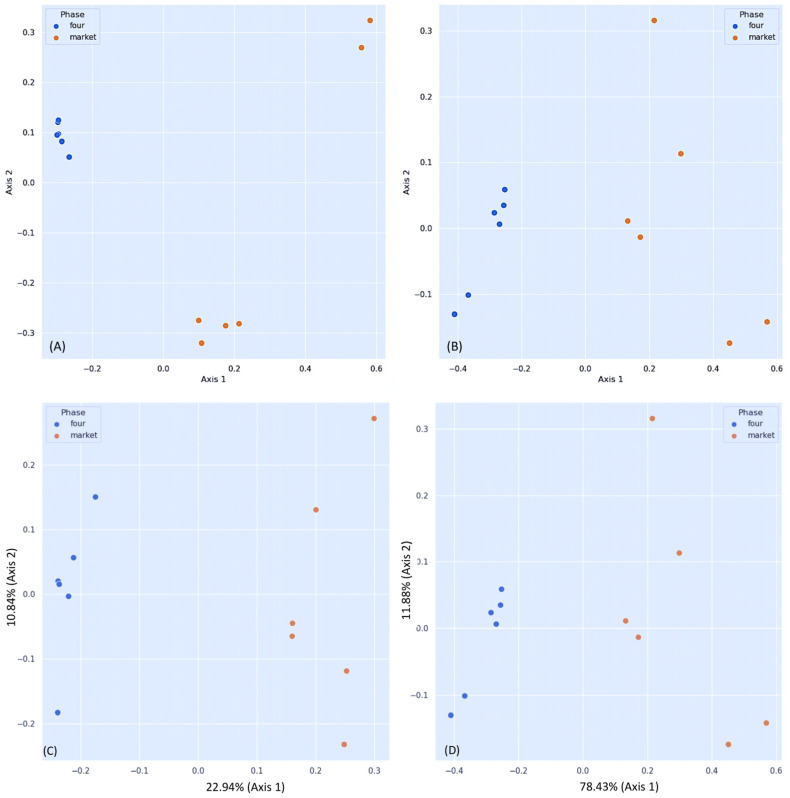
Principal Coordinate Analysis (PCoA) plots of fungal beta diversity. (**A**) Bray–Curtis dissimilarity indices; (**B**) Jaccard distance; (**C**) unweighted UniFrac distance, (**D**) weighted UniFrac distance. Statistics were calculated using pairwise PERMANOVA with 999 permutations.

According to the non-phylogenetic distance analyses Jaccard (PERMANOVA, pseudo-F = 3.119, *p* = 0.003) (Figure 2A) and Bray–Curtis (PERMANOVA, pseudo-F = 8.192, *p* = 0.006), we found significant differences between the groups (orchard vs. market) (Figure 2B). The diversity of the fungi between groups based on unweighted and weighted UniFrac metrics revealed significant differences (*p* = 0.03) between the orchard and the market (Table 2). When calculating the unweighted UniFrac distance as a phylogenetic index (PERMANOVA, pseudo-F = 3.994, *p* = 0.003), significant differences were found (Figure 2C). The market group samples formed a distinct cluster from the orchard samples, according to the PCoA map for the abundance of unweighted UniFrac distance. Furthermore, significant differences in beta diversity were detected when the weighted UniFrac distance was measured (PERMANOVA, pseudo-F = 22.2981, *p* = 0.003) (Figure 2D). Variable F1 (Axis 1) explained 78.43% of the total variance by loading the market samples, whereas variable F2 (Axis 2) explained 11.88% of the variance by loading the orchard samples. In addition, R values near 1.0 (0.99 and 0.93, respectively) suggest that there is a dissimilarity between groups based on ANOSIM and the unweighted and weighted UniFrac distance results. Similarly, the Bray–Curtis and Jaccard similarity results indicate dissimilarity between the groups (R values: 1.00 and 0.59, respectively). These results were in concordance with our previous DNA barcoding study on strawberry fruits, showing that the bacterial community of market group was divergent from the field group [17]. Like these findings, early metagenome surveys in strawberry showed a high beta-diversity of fungi in plant organs, but not in unripe and ripe fruits; the differences were related to the cultivar rather than the maturity stage [11,31]. It should be noted that the fruits used in this study were obtained from a conventional orchard (fungicidal treatments). However, there were significant differences in fungal diversity between the market and orchard groups, which means that upon harvest the fruits are more susceptible to contamination.

### 3.3. Taxonomic Assignment of Fungi

**Figure 3:**
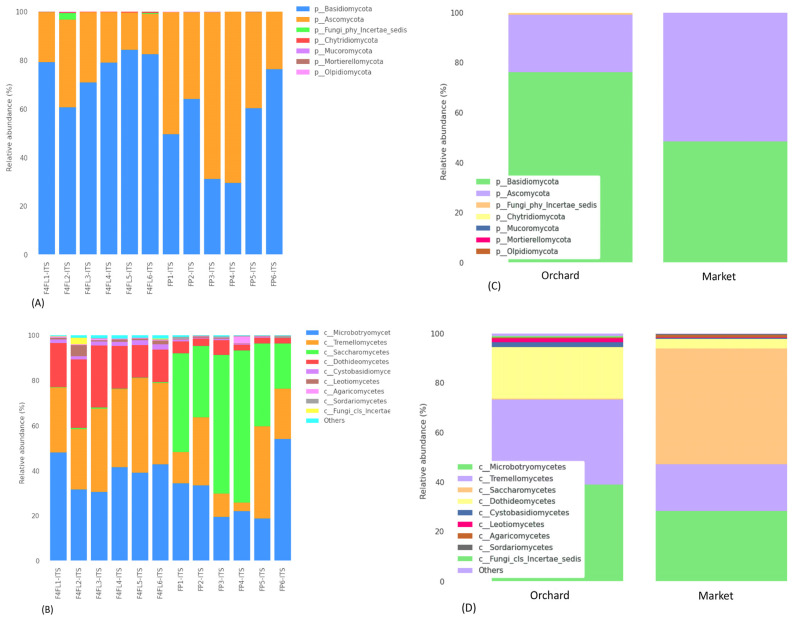
Relative abundance (%) of different fungal phyla and classes detected in strawberry fruits across the samples (**A**,**B**) and among the groups (**C**,**D**). Legend: F4L1-ITS: F4L6-ITS: fruits collected from the orchard; FP1-ITS:FP6-ITS: fruits purchased from the market. The “Other” category in this sum of all classifications with less than 0.50% abundance.

**Figure 4:**
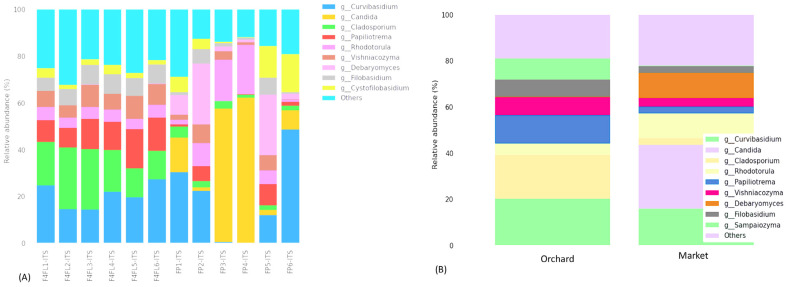
Relative abundance (%) of different fungal genera identified in strawberry fruits across the samples (**A**), and among the groups (**B**). Legend: F4L1-ITS: F4L6-ITS: fruits collected from the orchard; FP1-ITS:FP6-ITS: fruits purchased from the market. The “Other” category in this sum of all classifications with less than 0.50% abundance.

The taxonomic assignment of ASVs in fruits revealed 7 phyla and 31 classes. The fungal phyla were predominantly *Basidiomycota* (varies from 29.59 to 84.58%), *Ascomycota* (varies from 15.33 to 70.40%), Fungi-phy-Insertae-sedis (varies from 0.45 to 2.89%), as well as in lower abundance *Chytridiomycota*, *Mucoromycota*, *Mortierellomycota* and *Olpidiomycota* (Figure 3A). The most predominant classes were *Microbotryomycetes*, *Tremellomycetes*, and *Dothideomycetes* in the samples collected from the orchard, while *Saccharomycetes* and *Microbotryomycetes* were the most predominant in the market (Figure 3B). Between the groups, *Basidiomycota* was predominant in the orchard group, while *Ascomycota* was predominant in the market group (Figure 3C). Likewise, the most predominant classes among the groups were *Saccharomycetes* in the market group and *Microbotryomycetes* and *Tremellomycetes* in the orchard group (Figure 3D). In previous metagenomic analyses in different compartments of strawberry plants and ripe fruits, the fungal phyla were predominantly *Ascomycota*, *Mortierellomycota*, and *Basidiomycota*, and their prevalence was related to the cultivar and plant compartments [31]. Similarly, in the study of Jones et al. [33], *Ascomycota* followed by *Basidiomycota* and *Zygomycota* were the most abundant in strawberry fruits collected during the ripening stage from an orchard in the United Kingdom. However, no information was provided about the cultivar used. At the genus level, *Curvibasidium*, *Cladosporium*, and *Papilliotrema* were the most predominant in the orchard sample, while *Candida*, *Rhodotorula*, and *Debaryomyces* were the most prevalent in the market samples (Figure 4A,B). A recent study showed that endophytic yeasts such as *Curvibasidium* were detected in several fresh leafy vegetables and berry fruits [34]. Nonetheless, its role in the safety and quality of strawberry fruits was not investigated. Early research showed that *Cladosporium* spp. Are pathogens causing blossom blight in strawberries [35]. This pathogen causes green-gray sporulation on dead tissue and misshaped fruits [36]. A very recent study associated that some fungal species of the genera *Papiliotrema*, *Vishniacozyma*, and *Filobasidium* are responsible for the flavor formation of strawberries [37]. Microbiome surveys in strawberry plants and fruits showed that *Botrytis* and *Cladosporium* represented 70–99% of the relative abundance of all sequences [11]. The phyllosphere compartments contained the predominant fungal genera *Mycosphaerella*, unidentified *Capnodiales*, *Alternaria*, and unidentified *Helotiales.* The abundance of these genera varies depending on the strawberry cultivar. *Botrytis* was found in ripe fruits and had a similar distribution between the strawberry cultivars ‘Mara des Bois’ and ‘White annanas’ [31]. In the 26S ribosomal DNA study of Satitmunnaithum et al. [2] performed in ‘Tochiotome’ variety fruits obtained from a greenhouse field, the most abundant fungi were *Altenaria* spp., *Aspergillius* spp., *Cryptococcus* spp., and *Ustilago* spp. In another study, several fungal pathogens including *Alternaria alternata* (black leaf spot), *Botrytis caroliniana* (gray mold), and *Plectosphaerella cucumerina* (fruit root and collar rot) colonize healthy strawberry plants [38,39]. In the current investigation, the genus *Botrytis* and *Altenaria* were less prevalent (<0.2%) in the samples. Since the plants in previous studies were grown above 360 m and 877 m elevation [2,11], respectively, we speculate that the relative abundance of some species may be related to the growing conditions, above 2300 m elevation, where the soil is primarily volcanic and the storage conditions and to a lesser extent the variety. In addition, the difference in microbiome might be related to the differences on sampling seasonality. Furthermore, our metagenomic study indicates that there is no presence of *B. cinerea*, which is the causing agent of gray mold disease in almost all vegetable and fruit crops, including strawberry plants and post-harvest fruits [40]. We found *B. caroliniana* in all samples regardless of their origin. The lower abundance of these species could be explained by the fact that we selected intact fruits, with no visible spoilage at stage four. Based on ANCOM analysis, we found that the most differentially abundant fungal genera were *Hanseniaspora*, *Kurtzmaniella*, and *Phyllozyma* (Table 3). A volcano plot showing the ANCOM model W statistic is shown in Figure S2. *Hanseniaspora uvarum* (relative abundance of 0.33%) and *Kurtzmaniella cleridarum* (relative abundance of 0.23%) were the most common taxa in the samples from the market, while *Phyllozyma copromiscola* (relative abundance of 0.5%) was prevalent in the samples from the orchard. In both immature and ripe strawberries, taxa belonging to the *Hanseniaspora*, but not *Kurtzmaniella*, were previously found in lower abundance (0.1–0.13%) [31]. The *Kurtzmaniella* genus was described with a novel species *K. cleridarum* isolated from rotting wood, mushrooms, and fruits [41]. In a recent study, mature raspberry and strawberry fruits were shown to have a greater prevalence of *Saccharomycetales*, including *H. uvarum*, being among the fruits with the highest susceptibility to *Drosophila suzukii* [33]. *Phyllozyma* is a relatively new genus according to a recent taxonomy study [42]. Previous metagenomic surveys of strawberry plants and ripe fruits showed that the fungal microbiome varies with the strawberry cultivar and ripe stage [31]. However, the impact of *Phyllozyma* in fruit quality is not yet documented. Thus, we suggest that the mycobiome differences could be related to the cultivar, harvest season, geographic origin, and agriculture system.

### 3.4. Taxonomic Assignment Suggests Both Beneficial and Harmful Yeasts in Strawberry Fruits

**Figure 5:**
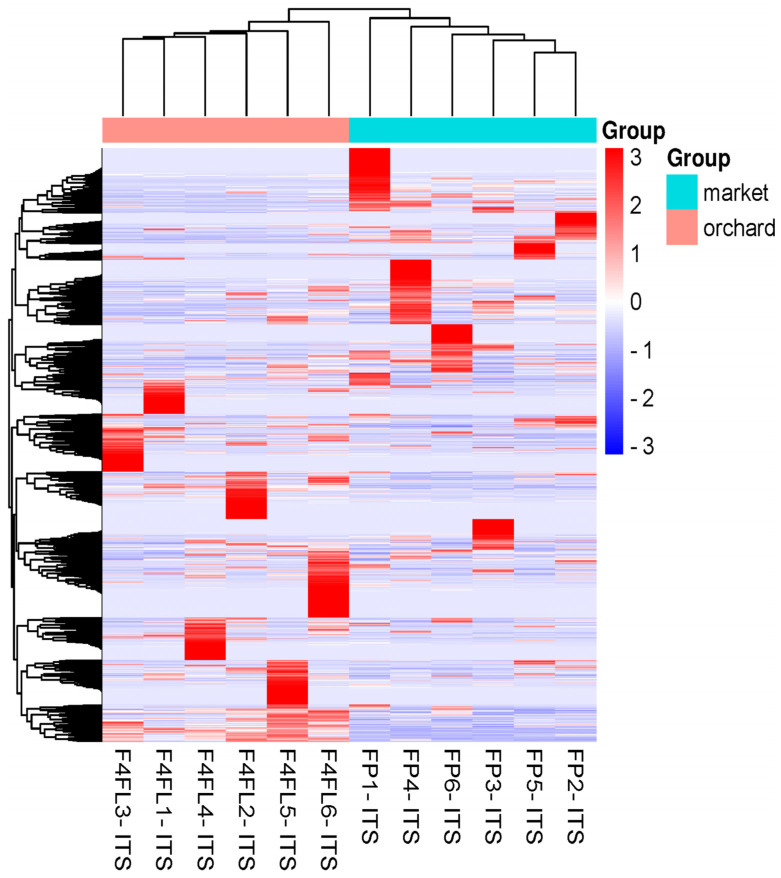
Heatmap and hierarchical clustering of the most abundant fungi at the genus level. *X*-axis contains the microbial genus that have been identified in the samples; on the *Y* axis are the different samples and experimental conditions that are being compared (groups); blue intense color: high abundance, pink color: low abundance.

**Figure 6:**
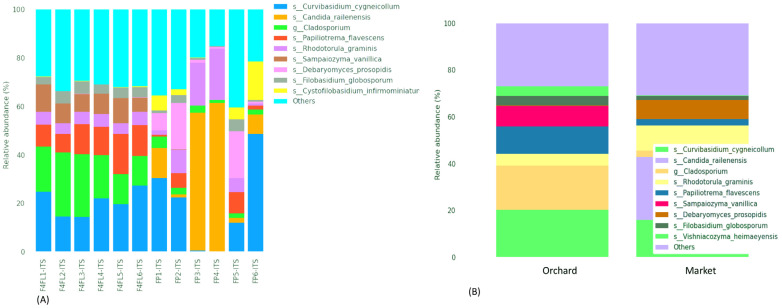
Relative abundance (%) of fungi species identified in strawberry fruits across the samples (**A**) and among the groups (**B**). Legend: F4L1-ITS: F4L6-ITS: fruits collected from the orchard; FP1-ITS:FP6-ITS: fruits purchased from the market. The “Other” category in this sum of all classifications with less than 0.50% abundance.

Fruits can be more storable and less susceptible to disease if their microbiomes are healthier and diverse. Early research indicates that during developmental stages, significant microbial fractions are transferred between different plant compartments [31]. Furthermore, as documented by Yan et al. [43], yeasts can colonize fruit surfaces for a prolonged duration; they can also inhibit the germination of pathogen spores and the length of the germ tube [44], induce a variety of defense-related enzyme activities that trigger host defense mechanisms, and assist in the biosynthesis of antimicrobial compounds [45]. Based on the abundance information of the main genera/species of all samples, a heatmap was drawn and the similarity and difference were shown (Figure 5). Similarly, the groups shared 91 (22.8%) genera and 128 (26.4%) species (Figure S3). Endophytic yeasts such as *Curvibasidium cygneicollum* were prevalent in strawberry samples regardless of the origin, except the market samples FP3-ITS and FP4-ITS (Figure 6A,B). *C. cygneicollum* was among the most common Basidiomycetes yeasts detected in stored fruits [46]. Interestingly, the predominant yeast species, such as *Candida railenensis*, was detected in fruits only from the market. Their relative abundance varies with the sample from 1.3% to 61.42%. C. *railenensis* was detected in grape berries and was the weakly ascomycetes associated with the fermentation to be used in the production of ice wine [47]. Likewise, *Debaryomyces prosopidis* was detected in samples from the market. From early analyses of fungal diversity in strawberries, these yeast species were not documented in ripe strawberry [11,31]. Thus, we suggest that might have something to do with the geographical origin, season, and the cultivar. *Rhodotorula graminis* (relative abundance varying from 1.7% to 21.18%) and *Papiliotrema flavescens* (relative abundance varying from 1.58% to 16.55%) were detected in all samples regardless of the origin, while *Debaryomyces prosopidis* was detected in samples from the market only, their relative abundance varying with the sample (0.80–19.23%) (Figure 6A,B). Previous studies indicate that *R. graminis* produces beta-carotene, torulene, and torularhodin to counteract ROS-induced cellular damage [48]. Additionally, *Filobasidium globosporum* was predominant in the samples from the orchard. Furthermore, although in lower abundance (0.9%) species of *Colletotrichum* were predicted in market-derived samples. Among them, *C. acutatum*, known as the leading cause of anthracnosis [49,50] in strawberries, was not detected in this study. Finally, a phylogenetic tree was built to reveal evolutionary relationships and biological diversity in a sequence data set (Figure S4).

### 3.5. Clinically Related Yeasts Were Detected in Strawberries Fruits from Market

**Figure 7:**
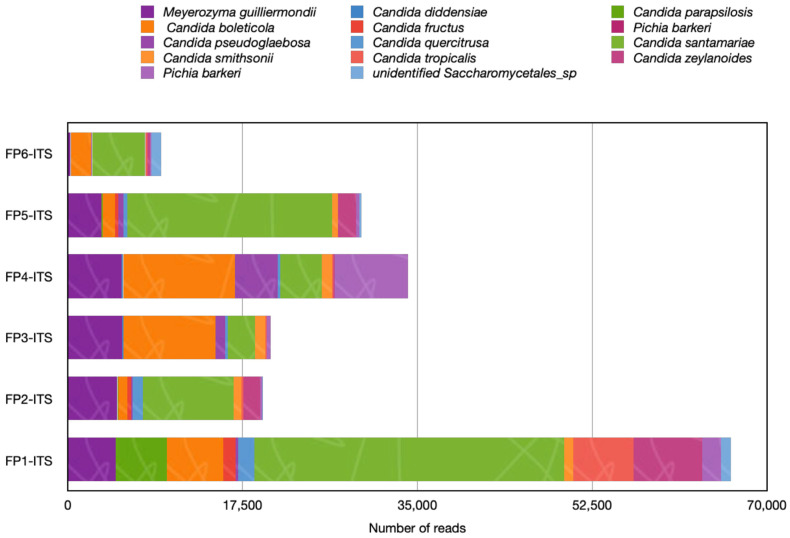
Clinical related yeasts species detected in samples purchased from the market (expressed as number of reads). Legend: FP1-ITS: FP6-ITS: fruits purchased from the market.

The widespread presence of yeasts in natura, including soil, plant substrates, the atmosphere, and geographic zones, as well as humans’ close association with specific yeast taxa, are the primary causes of the emergence of mycoses and allergic diseases [51]. There are nine genera of clinically significant yeasts that are regularly isolated from naturally occurring soil-plant substrates: *Saccharomyces*, *Rhodotorula*, *Geotrichum*, *Meyerozyma*, *Pichia*, *Candida*, *Trichosporon*, *Mallasezia*, and *Cryptococcus* [51]. The most common mycological conditions related to yeast are dermatitis, alveolitis, rhinitis, allergic bronchopulmonary mycosis, and bronchial asthma [52]. *Cryptococcus albidus* was previously detected in strawberry fruits stored at 15 °C with impact on the fruit quality [2]. Interestingly, in the current study, we detected *Cryptococcus frias* in both orchard and market fruits with a relative abundance of 0.5% in the market samples and 8.89% in the samples from the orchard. No studies mention the detection of these species in fruits. Early research associate *C. frias* with the glacial biomes of Patagonia [53]. Members of the *Candida* pathogenic clade, such as *C. albicans*, *C. parapsilosis*, *C. tropicalis*, and recently *C. auris*, were found in commensal environments including fruits [54]. *C. albicans* is known as an opportunistic yeast pathogen that causes infections in immunosuppressed patients, including skin infections in diabetic patients and thrush in infants [55]. In this metagenomic analysis, several clinically associated *Candida* yeasts, but not *C. albicans* and *C. auris*, were detected in the samples from the market (Figure 7). Among yeasts, the most common were *Meyerozyma guilliermondii*, *C. parapsilosis*, and *C. santamariae*, their abundance varied by sample stand. We suggested that the absence of these species in the orchard samples could indicate environmental contamination; however, more research is required to fully understand these concerns. The study by Abdelfattah and colleagues [11] did not report any species of *Candida* in strawberries. This may be connected to the fact that no fruit samples from the market were considered for the study. In addition, the geographical location and the plant variety might influence the fungal diversity. Nonetheless, *C. parapsilosis* was detected on the skin of fresh fruits and showed antibiotic resistance [56]. Whether these cultures have evolved to survive as endophytes remain unknown. It is crucial to recognize that the surfaces of fruits can harbor pathogenic yeasts, particularly drug-resistant strains that could potentially spread to humans [54]. Previously, *M. guilliermondii* and *C. parapsilosis* were detected in commercial fruits and vegetables in Russia [46]. All fruit samples have been shown to contain a high number of yeasts, although no indicator of these microorganisms is specified in the hygienic requirements legislation for the safety of fruit products [47]. However, our findings did highlight the need for further microbiological crop control, as well as the creation of new criteria to determine the presence of yeast that are clinically relevant in fresh products.

## 4. Conclusions

Taken together, this is the first study comparing the diversity and structure of fungal communities associated with strawberries obtained from a volcanic orchard and commercial market. The fungal communities vary amongst the groups, as in the market group, along with several beneficial taxa that might improve the fruit quality, an increase in potentially pathogenic taxa was observed. Based on the microbiome composition analysis, *Hanseniaspora*, *Kurtzmaniella*, and *Phyllozyma* were the most differentially abundant fungal genera. *Curvibasidium cygneicollum*, an endophytic yeast, was found in both groups, while *Candida railenensis* and *Debaryomyces prosopidis* were found only in fruits obtained from the market; their relative abundance varies with the sample. Additionally, an unexpected presence of multiple clinically related yeasts, including *Meyerozyma guilliermondii* and *Candida parapsilosis*, were detected in the market samples. More research should examine the relationship between the mycobiome and bacteriome in ready-to-eat fruits in the context of safety and quality. Further work implies the need to develop strategies to identify the presence of clinically significant yeasts in fresh products before sale, in addition to measurements to increase the safety.

## Figures and Tables

**Table 1:** Shannon species diversity in strawberry fruits.

Sample Origin	Sample ID	Number Reads Passing Quality Filtering	% Reads Classified to Genus	Shannon Species Diversity	Number of Species Identified
Orchard	F4FL1-ITS	402,166	88.15%	2.47	582
	F4FL2-ITS	413,205	83.63%	2.47	563
	F4FL3-ITS	362,685	85.13%	2.38	567
	F4FL4-ITS	374,315	86.46%	2.56	598
	F4FL5-ITS	473,020	82.99%	2.61	628
	F4FL6-ITS	330,732	84.94%	2.60	783
Market	FP1-ITS	536,563	96.52%	3.05	848
	FP2-ITS	457,487	92.27%	2.78	699
	FP3-ITS	479,798	97.05%	2.54	644
	FP4-ITS	546,063	96.72%	2.26	737
	FP5-ITS	395,658	89.55%	2.85	587
	FP6-ITS	241,369	92.60%	2.45	684

**Table 2:** Beta-diversity metrics in strawberry fruits. The significance was determined through 999 Monte Carlo permutations; the values were considered significant when *p* < 0.05.

Metrics	Pseudo-F *	*p*-Value	q-Value
Bray–Curtis dissimilarity	8.19245731	0.006	0.006
Jaccard distance	3.11991142	0.003	0.003
Unweighted_unifrac distance	3.94475851	0.003	0.003
Weighted_unifrac distance	22.2981783	0.004	0.004

* Pseudo-F value [32].

**Table 3:** Abundance (%) features by group.

Taxon	W	Reject Null Hypothesis	% of Abundance									
			0	25	50	75	100	0	25	50	75	100
			Orchard				Market					
*Phyllozyma* spp.	369	TRUE	264	533.25	671	732.25	1057	1	1	1	1	1
*Kurtzmaniella* spp.	367	TRUE	1	1	1	1	1	219	261.75	1128	2154.75	7074
*Hanseniaspora* spp.	348	TRUE	1	1	1	1	1	119	134	387.5	652.25	2136

## Data Availability

Raw sequence data were deposited in the National Collection of Biotechnology Information under Bioproject ID PRJNA1085588 on 11 March 2024. (https://www.ncbi.nlm.nih.gov/sra/PRJNA1085888, accessed on 14 February 2024).

## Supplementary Materials

The following supporting information can be downloaded at: https://www.mdpi.com/article/10.3390/foods13081175/s1, Figure S1: Illustration of rarefaction curves. Figure S2: A volcano plot showing the ANCOM model W statistic in strawberries. Figure S3: Venn diagram showing the number and percentage of shared fungi (A) genus and (B) species level. Figure S4: Phylogenetic tree derived from ITS2 sequence data showing the position of the most abundant fungal taxon. Table S1. DADA2 statistics of input and filtered reads.
