# Supplementary material for: Fungal Mycobiome of Mature Strawberry Fruits (Fragaria x ananassa Variety ‘Monterey’) Suggests a Potential Market Site Contamination with Harmful Yeasts
Source: Foods. 2024 Apr 12;13(8):1175. doi: 10.3390/foods13081175 (PMC11049331; doi:10.3390/foods13081175)
Supplement: Supplementary file 1 [file foods-13-01175-s001.zip › foods-2943337-supplementary.pdf]

Supplementary materials

Fungal Mycobiome of Mature Strawberry Fruits (*Fragaria x ananassa* Variety 'Monterey') Suggests a Potential Market Site Contamination with Harmful Yeasts

Gabriela N. Tenea *, Pamela Reyes and Diana Molina

Biofood and Nutraceutics Research and Development Group, Faculty of Engineering in Agricultural and Environmental Sciences, Universidad Técnica del Norte, 100150 Ibarra, Ecuador

* Correspondence: gntenea@utn.edu.ec

Figure S1: Illustration of rarefaction curves. Legend: F4L1-ITS: F4L6-ITS: fruits collected from the orchard; FP1-ITS:FP6-ITS: fruits purchased from the market.

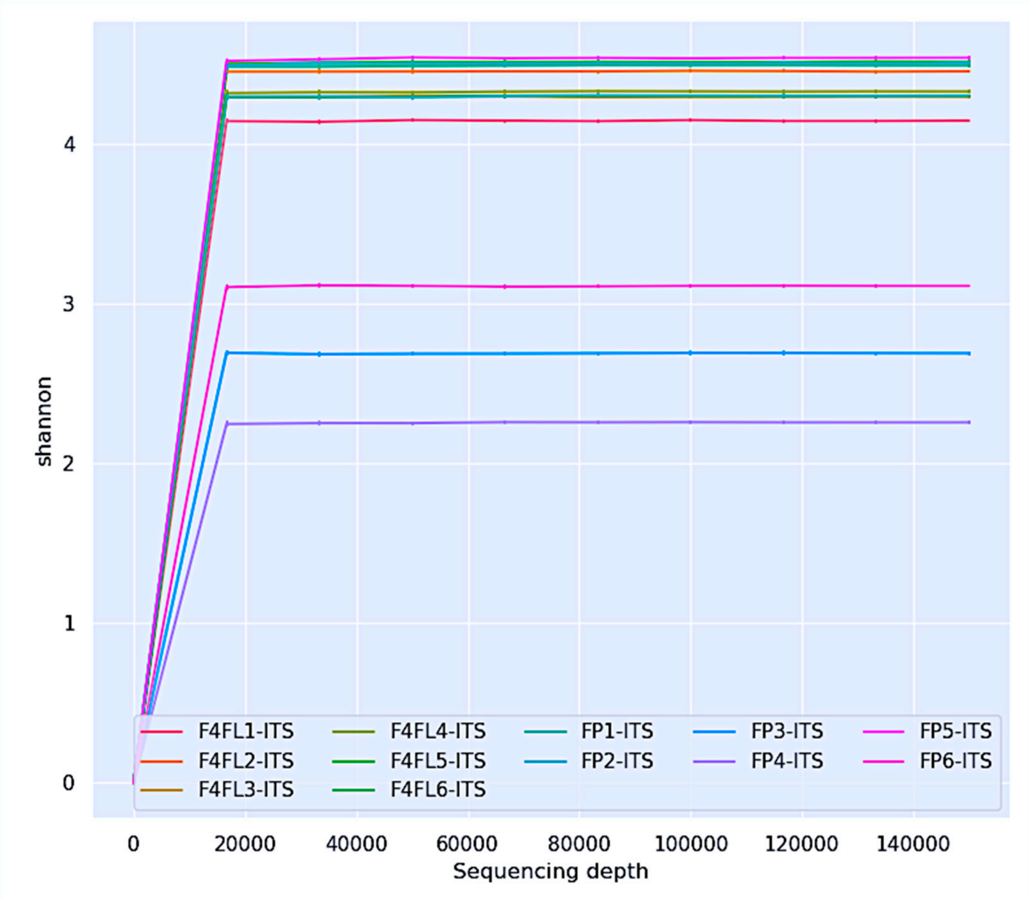

Figure S2: A volcano plot showing the ANCOM model W statistic.

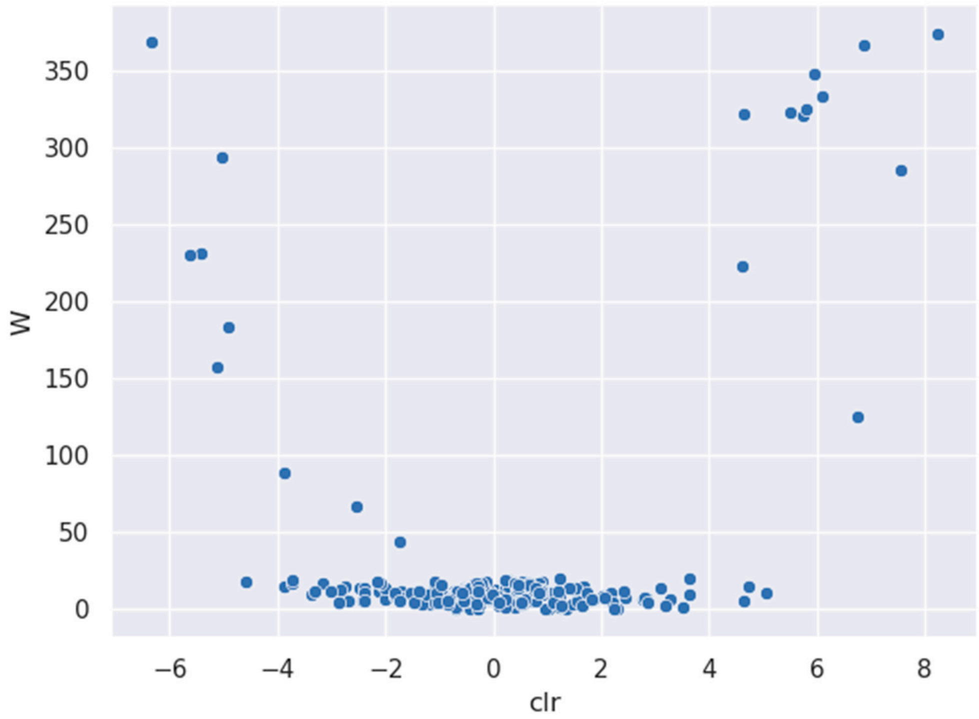

Figure S3: Venn diagram showing the number and percentage of shared fungi (A) genus, and (B) species level.

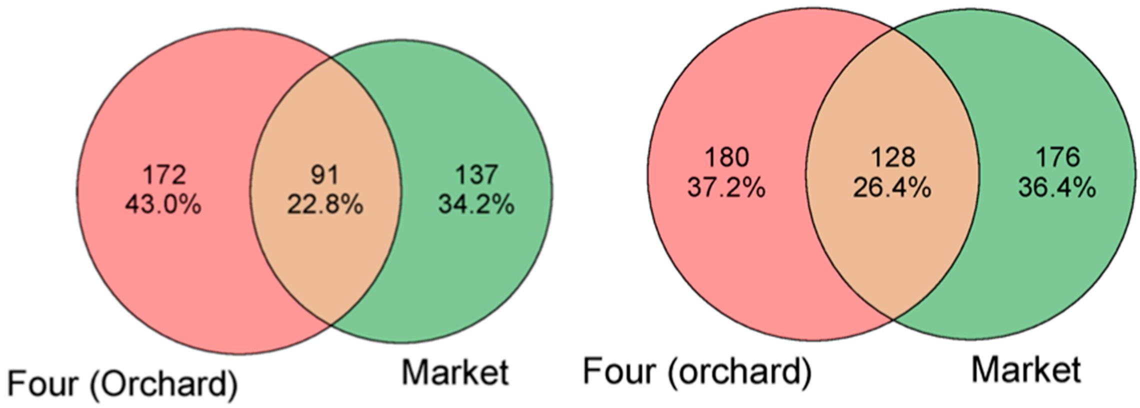

Figure S4: Phylogenetic tree derived from ITS2 sequences data showing the position of the most abundant fungal taxon. Color nodes in the tree are features (taxonomic annotation to the family level). Internal nodes where all descendants have the same feature metadata value are themselves considered to have that value. Bar plots are the groups metadata.

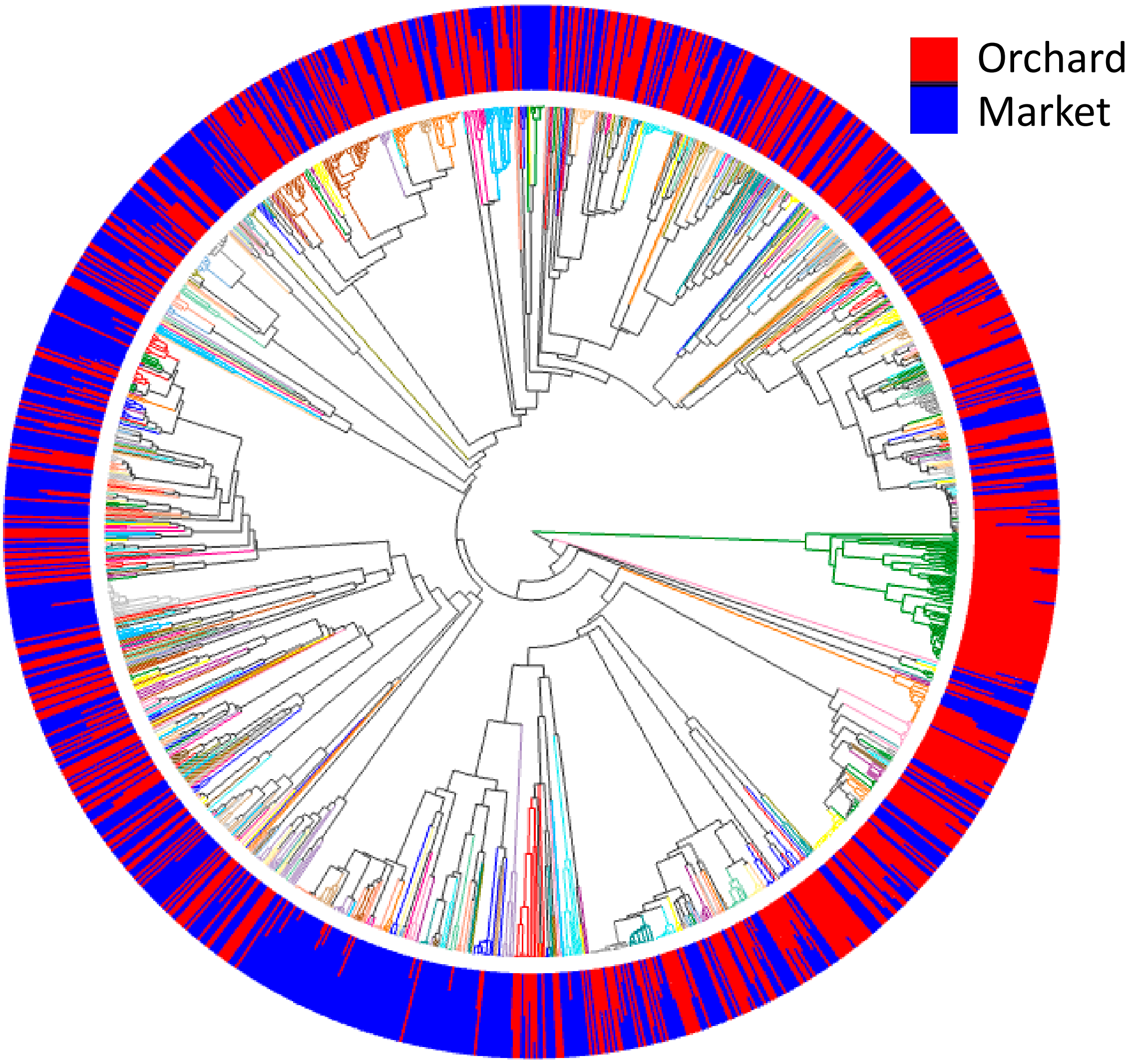

Table S1. DADA2 statistics of input and filtered reads.

sample-id	Input	filtered	percentage of input passed filter	denoised	merged	percentage of input merged	non-chimeric	percentage of input non-chimeric
**F4FL1-ITS**	402166	274966	68.37	274294	249699	62.09	249157	61.95
**F4FL2-ITS**	413205	259061	62.7	258323	234064	56.65	233380	56.48
**F4FL3-ITS**	362685	251092	69.23	250745	224918	62.01	224724	61.96
**F4FL4-ITS**	374315	249823	66.74	249554	231034	61.72	230731	61.64
**F4FL5-ITS**	473020	294291	62.22	293716	272461	57.6	271758	57.45
**F4FL6-ITS**	330732	181704	54.94	181389	158520	47.93	158184	47.83
**FP1-ITS**	536563	347337	64.73	346999	321390	59.9	320593	59.75
**FP2-ITS**	457487	323935	70.81	323401	306142	66.92	305628	66.81
**FP3-ITS**	479798	332582	69.32	332384	305789	63.73	305233	63.62
**FP4-ITS**	546063	401669	73.56	401393	377459	69.12	376760	69
**FP5-ITS**	395658	278264	70.33	278032	265122	67.01	265001	66.98
**FP6-ITS**	241369	158184	65.54	157860	151598	62.81	151217	62.65

F4L1-ITS: F4L6-ITS-fruits collected from the orchard (ripe phase four); FP1-ITS: FP6-ITS: fruits purchased from the market (ripe phase four).
